# Does metformin improve *in vitro* maturation and ultrastructure of oocytes retrieved from estradiol valerate polycystic ovary syndrome-induced rats

**DOI:** 10.1186/s13048-015-0203-x

**Published:** 2015-11-14

**Authors:** Fakhroddin Mesbah, Mohsen Moslem, Zahra Vojdani, Hossein Mirkhani

**Affiliations:** Department of Anatomical Sciences, Shiraz University of Medical Sciences, Shiraz, 71348-53185 Iran; Embryonic Stem Cell Lab, Shiraz University of Medical Sciences, Shiraz, Iran; Department of Pharmacology, School of Medicine, Shiraz University of Medical Sciences, Shiraz, Iran; Medicinal and Natural Products Chemistry Research Center, Shiraz University of Medical Sciences, Shiraz, Iran

**Keywords:** Polycystic ovary syndrome, *In vitro* oocyte maturation, Metformin

## Abstract

**Background:**

Metformin decreases polycystic ovary syndrome (PCOS) symptoms, induces ovulation, and may improve developmental competence of *in vitro* oocyte maturation. This study was designed to define the effects of metformin on the characteristics of *in vitro* oocyte maturation in estradiol valerate (EV) PCOS-induced rats.

**Methods:**

Forty-five adult female Sprague–Dawley rats were randomly divided into control; sham and PCOS-induced (treated by a single dose of estradiol valerate, 4 mg/rat, IM) groups. The body weight was measured weekly for 12 weeks. At the end of week 12, the serum levels of testosterone, estrogen, progesterone, LH, and FSH and blood glucose of all the rats were measured. About 380 cumulus oocyte complexes (control, 125; sham, 122; PCOS-induced rats, 133) were incubated in Ham’s F10 in the absence and/or presence of metformin (M 5^−10^) for 12, 24, 36, and 48 h. The cumulus cells expansion and nuclear and cytoplasmic maturation of the oocytes was evaluated using 1 % aceto-orcein staining, and transmission electron microscopy (TEM).

**Results:**

No significant differences were observed in the body weight of the rats. The serum level of testosterone was reduced, and progesterone and LH were significantly increased in the PCOS-induced rats (*p* < 0.05). However, no significant differences were observed in the serum levels of estrogen and FSH among the groups. Blood glucose level was higher in the PCOS-induced rats than control, (*p* < 0.01). The expansion of cumulus cells was observed in the metformin-treated oocytes. The oocytes retrieved from PCOS-induced rats show a stage of meiotic division (GVBD, MI, A-T, and MII) in 57.12 % of metformin-untreated and fairly significantly increased to 64.28 % in metformin-treated oocytes, (*p* < 0.05), but no differences were observed in the MII stage within groups. The redistribution of some cytoplasmic organelles throughout the ooplasm, particularly the peripheral cortical granules, was defined in the metformin-treated oocytes.

**Conclusions:**

Single dose of EV can creates a reversible PCO adult rat model. Metformin enhances the COCs to initiate meiotic resumption at the first 6 h of IVM. In our study the metformin inability to show all aspects of *in vitro* oocyte maturation and may be resulted from deficiency of EV to induce PCOS.

## Background

Polycystic ovary syndrome is one of the major causes of anovulatory infertility in women. The etiology and pathology of PCOS are still questioned, but imbalances of testosterone, estrogen, progesterone, LH and FSH, obesity, insulin resistance, and hyperinsulinemia are crucial factors for ovarian hyperandrogenism and chronic anovulation [1]. In addition to infertility, patients with PCOS are at a risk of miscarriage [2], which has been reported during the first trimester of pregnancy in approximately 30 to 35 % of patients [3].

The protocols proposed for the treatment of PCOS include a diet plan, exercise and physical activity, drug treatments, and surgical procedures [4]. Drug treatments include the administration of metformin, glitazones, spironolactone, estrogen, and clomiphene citrate [5]. In recent years, metformin alone or in combination with other drugs is used to treat PCOS. Metformin, a biguanide antihyperglycemic drug, is utilized to treat type 2 diabetes. In women with PCOS associated with anovulation and resistance to clomiphene citrate, the administration of clomiphene citrate in combination with metformin satisfactorily increases the ovulation and pregnancy rate [6].

Many studies have reported that metformin can induce oocyte maturation and improve oocyte quality [7]. However, the role of metformin in promoting *in vitro* oocyte maturation remains unknown. The controversial effects of metformin on *in vitro* maturation (IVM) of oocytes have been demonstrated. Lee et al. demonstrated the effects of metformin on the *in vitro* developmental potential of porcine oocytes and embryos; further, they clearly asserted that metformin augments the actions of insulin as an insulin-sensitizing agent on the cytoplasmic aspect of oocyte maturation and the preimplantation embryonic development during *in vitro* production [8]. In addition, the administration of metformin in combination with oral contraceptives to women with PCOS can increase the probability of *in vitro* oocyte maturation in relation with miscarriage rate [9]. In contrast, Tosca et al. demonstrated that metformin activates adenosine monophosphate-activated protein kinase (AMPK), which inhibits the germinal vesicle breakdown (GVBD) in cumulus oocyte complexes (COCs) and most COCs arrested at the germinal vesicle (GV) stage, but not denuded bovine oocytes, [10]. Bilodeau-Goeseels et al. also reported that metformin, as an activator of AMPK, inhibited GVBD in bovine cumulus-enclosed oocytes and denuded oocytes and that AMPK might play contradictory roles in the regulation of bovine and murine oocyte maturation, [11].

Despite the ethical and technical restrictions on human experimentations, so far, the animal models that can display all features of human PCOS has not been well introduced. On the other hand, the effects of supplements on the *in vitro* oocyte maturation to be clearly determined, when all the main characteristics of PCOS are observed. Sabatini et al. revealed that metformin reduced the IVM of oocytes of the wild type and leptin deficient transgenic (ob/ob) mice models, but not in leptin receptor mutant mice (db/db) models, [12]. The *in vitro* maturation of oocytes consists of two principle features: nuclear and cytoplasmic maturation [8]. Thus far, the precise role of metformin in promoting all the aspects of oocyte maturation in animal models of PCOS has not been identified. The purpose of this study was that, despite the fact that EV PCOS-induced rat model lack some endocrine and metabolic features, whether metformin contributes to the quality of the *in vitro* maturation of oocytes? Therefore, the present study was designed to investigate the effects of metformin as an additive supplement on nuclear and cytoplasmic maturation as well as the expansion of cumulus cells as the third criterion of oocyte maturation in in EV PCOS-induced rats.

## Methods

### Experimental design

Forty-five adult female Sprague–Dawley rats (170–230 g) were randomly selected from the animal house of Shiraz University of Medical Sciences (SUMS). The animals were reared under standard conditions (12 h of darkness; 12 h of light; at 23 ± 2 °C), with *ad libitum* access to food and tap water. The SUMS Ethics Committee approved all the animal procedures. The animals were randomly classified into three groups: PCOS-induced, which received a single dose of EV, 4 mg/0.4 mL of olive oil/rat, IM, to induce PCOS; sham, received only olive oil, 0.4 mL/rat, IM; control, received no drug. This study was designed for a period of 12 weeks, and all the animal groups were kept under standard conditions in separate cages.

### Measurement of weight, sex hormones and blood glucose

For defining the characteristics of the animal model of PCOS, the body weight of all the rats was measured once a week for 12 weeks on the same day at the same time; the serum levels of testosterone, estrogen, progesterone, LH, and FSH and blood glucose were also assessed (radioimmunoassay), at the end of week 12.

### Histological study

To recognize the presence of cystic follicles in PCOS-induced rats, paraffinzed blocks of the ovaries were prepared. The blocks were cut into 5-μm-thick sections, stained with hematoxylin-eosin, and observed by a light microscope.

### Oocyte collection and *in vitro* maturation

After 12 weeks, the rats were sacrificed and the ovaries excised to acquire COCs. The COCs with a homogeneous ooplasm and at least two layers of cumulus cells were selected for IVM. The COCs of each group were divided into two subgroups and incubated in Ham’s F10 (Cat. No. T 071–01) supplemented with 2.1 mg of sodium bicarbonate/mL, 75 μg of penicillin G/mL, 75 μg of streptomycin/mL and 5 % human serum albumin, in the absence and/or presence of metformin (M 5^−10^) for 12, 24, 36, and 48 h. The concentration of metformin (M 5^−10^) was assigned based on the results of Lee et al. but not different concentration, because they have demonstrated that different concentrations of metformin alone does not affect the *in vitro* maturation of oocyte [8]. One of the subgroups of oocytes was stained with an aceto-orcein (1 %) solution to define nuclear maturation as the assortment of GV, GVBD, metaphase I (MI), anaphase-telophase (A-T), and MII, and as degenerated and non-detectable by using a light microscope [13, 14]. Cultured denuded oocytes were placed in the center of glass slide, covered and compressed gently with a cover slid which attached to the glass slid by a paraffin-vaseline wax bar on each corner of cover slid to hold and fixed them in acid acetic and methanol (1:3, v/v) for 24 h. Oocytes were stained with 1 % fresh aceto-orcein (1 % orcein in 45 % glacial acetic acid) by pushing aceto-orcein solution between glass slid and cover slid using insulin syringe, and immediately observed by light microscope. To prepare a 1 % aceto-orcein solution, 55 mL of glacial acetic acid was boiled and poured over 1 g of orcein powder. The solution was cooled, and 45 mL of distilled water was added to it.

### Evaluation of the expansion of cumulus cells

In both subgroups of oocytes, the expansion of cumulus cells was evaluated using a modification of the method described by Nandi et al. [15] and was categorized as follows: complete expansion (almost all cumulus cells dispersed around the oocytes in a colloidal matrix), semi expansion (the cumulus investment had been initiated for the expansion and was partially dissociated), no expansion (none of the cumulus cells were expanded and they were adherent to the zona pellucida), and degeneration.

### Oocyte preparation for transmission electron microscopy

The other subgroup of oocytes was fixed overnight in Karnovsky’s solution (2.5 % glutaraldehyde and 2 % paraformaldehyde in 0.1-M sodium cacodylate buffer) and post-fixed in 1 % OsO_4_ in 0.1-M sodium cacodylate buffer for 90 min. The samples were then dehydrated by passing them through an ethanol series, embedded in Epon 812 (TAAB, Aldermaston Berkshire, UK), and finally sectioned into semi-thin (1-μm-thick) slices. The sections were mounted on glass slides and stained with toluidine blue; they were examined using a light microscope. The tissue blocks were retrimmed and ultrathin (60–90 nm) sections were taken. These sections were collected on copper grids, stained with uranyl acetate for 15 min and lead citrate for 12 min at room temperature, and examined using TEM (Philips CM10, Amsterdam, Netherlands).

### Statistical analysis

To analyze the data related to the weight and the serum hormone levels, SPSS ver. 15 (SPSS Inc., Chicago, IL, USA) was used, and the weights of the rats were compared by mixed model analysis, and the serum level of hormones with one-way analysis of variance and post-hoc least significant difference. A chi-squared test was used to compare the rate of cumulus cell expansion and that of *in vitro* COCs maturation within the groups.

## Results

### Measurement of weight, sex hormones and blood glucose

The gain in body weight of the rats within the groups showed a growing trend, the mean (±SD) of the body weight of the rats from week 1 to week 12 increased from 186.20 ± 6.40 to 205.06 ± 3.50 g in the control group, from 186.60 ± 4.53 to 205.60 ± 2.10 g in the sham group, and from 170.26 ± 5.67 to 192.13 ± 2.10 g in the PCOS-induced group, although no significant differences were observed in the body weight of the rats.

The serum level of testosterone decreased, while the progesterone and LH significantly increased in the experimental rats (*p* < 0.05). No significant differences were observed in the estrogen and FSH levels in the three groups (Table 1). Blood glucose level was significantly increased the PCOS-induced (184.40 ± 11.03 mg/dL) vs control (154.40 ± 13.52 mg/dL) rats, (*p* < 0.01).

**Table 1:** Serum levels of testosterone, estrogen, progesterone, FSH, and LH in rats

a	Control	Sham	PCOS
	(15 rats)	(15 rats)	(15 rats)
Testosterone (ng/mL)	0.180 ± 0.068	0.180 ± 0.057	0.130 ± 0.031^a)^
Estrogen (pg/mL)	11.120 ± 1.460	10.970 ± 0.470	11.570 ± 0.870
Progesterone (pg/mL)	50.990 ± 9.974	50.770 ± 6.678	56.700 ± 4.400^a)^
FSH (ng/mL)	3.140 ± 0.350	3.790 ± 0.310	3.230 ± 0.470
LH (ng/mL)	0.160 ± 0.010	0.200 ± 0.030	0.760 ± 0.050^a)^

*PCOS* polycystic ovary syndrome

^a)^Significant differences from the control and sham groups (*p* < 0.05)

### Histological observations

The histological features of the ovaries under the light microscope showed cystic and relatively degenerated follicles in the PCOS-induced rats (data not shown here and published previously as mention and cited in discussion section).

### *In vitro* oocyte maturation

**Fig. 1:**
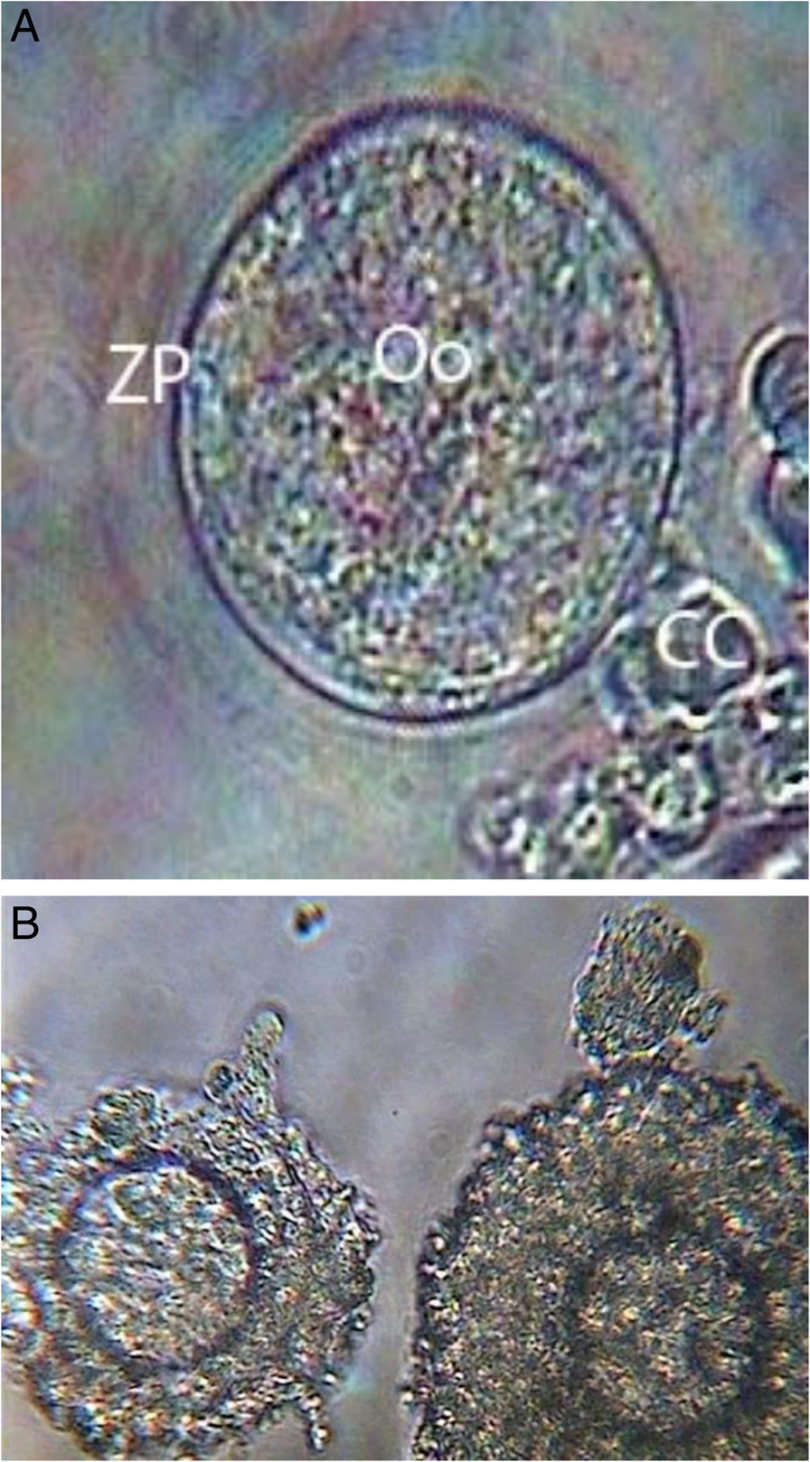
Cumulus oocyte complex expansion, (**a**) expanded COCs, cultured for 48 h in the presence of metformin in PCOS- induced (400×); (**b**) Non-expanded COCs, cultured for 24 h in the absence of metformin from the control rats (200×). ZP, zona pellucida; Oo, oocyte

Out of the 380 COCs cultured in all groups for 12, 24, 36, and 48 h, the cumulus cells of 53.03 % and 32.83 % of the COCs in the PCOS-induced rats in the metformin-treated and-untreated groups had expanded completely (Fig. 1a), 30.30 % and 25.374 % had semi-expanded, 15.15 % and 34.32 % had not expanded (Fig. 1b), and 1.51 % and 7.46 % had degenerated, respectively (Table 2). The cumulus cells in 64.21 % of the COCs had completely expanded and/or semi-expanded in the absence of metformin, and had increased to 88.94 % in the presence of metformin in all the rats. In addition, in the PCOS-induced rats, the percentage of completely expanded and semi-expanded cumulus cells increased from 58.20 to 83.33 %. The total proportion of COCs with no expansion of the cumulus cells decreased from 30.00 % in the absence of metformin to 10.00 % in the presence of metformin. These results demonstrate that the rate of complete expansion and semi-expansion of the cumulus cells increased significantly, and the percantage of unexpanded cumulus cells decreased significantly in the presence of metformin (*p* < 0.05).

**Table 2:** Expansion of cumulus cells of rat cumulus oocyte complexes in control, sham, and PCOS groups in the absence or presence of metformin

Groups	Without metformin				With metformin				
	Control	Sham	PCOS	Total	Control	Sham	PCOS	Total	Total
	*N* (%)	*N* (%)	*N* (%)	*N* (%)	*N* (%)	*N* (%)	*N* (%)	*N* (%)	*N* (%)
Expansion	25 (40.32)	25 (40.98)	22 (32.83)	72 (37.98)	34 (53.96)	35 (57.37)	35 (53.03)^a)^	104 (54.73)^a)^	176 (46.31)
Semi expansion	16 (25.80)	17(27.86)	17 (25.37)	50 (26.31)	22 (34.92)	23 (37.70)	20 (30.30)	65 (34.21)^a)^	115 (30.26)
No expansion	19 (30.65)	15 (24.59)	23 (34.32)	57 (30.00)	7 (11.11)	2 (3.27)	10 (15.15)^a)^	19 (10.00)^a)^	76 (20.00)
Degeneration	2 (3.23)	4 (6.55)	5 (7.46)	11 (5.78)	0 (00.00)	1 (1.63)	1 (1.51)^a)^	2 (1.05)^a)^	13 (3.43)
Total	62	61	67	190	63	61	66	190	380

*PCOS* polycystic ovary syndrome

^a)^Significant differences from the absence of metformin (*p* < 0.05)

**Fig. 2:**
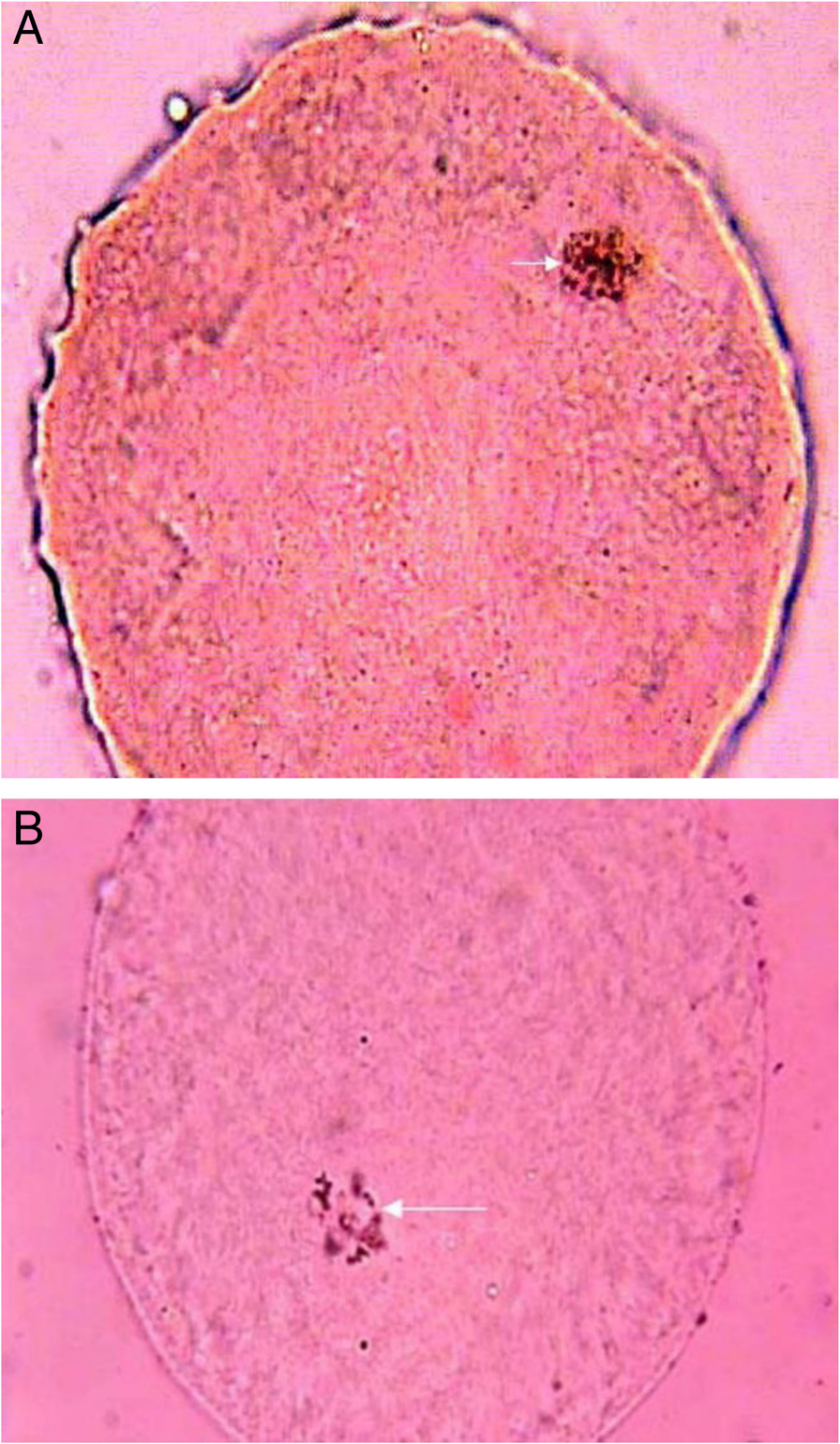
Light micrographs of nuclear maturation of oocytes cultured for, (**a**) 48 h in the presence of metformin at germinal vesicle breakdown (*arrow*) from the control; (**b**) 12 h in the presence of metformin at meiosis I (*arrow*) from the PCOS-induced rats. Aceto-orcein staining (1000×)

The nuclear maturation of oocytes was evaluated in the three groups: of 172 oocytes, 21 oocytes were in GV, 34 in GVBD (Fig. 2a), 37 in MI (Fig. 2b), 10 in A-T, 28 in MII, and 13 in degeneration; 29 were indistinguishable (Table 3). As mention in table 3, totally, 61.62 % of COCs show one of the stages of meiotic division (sum of GVBD, MI, A-T, and MII) in the absence of metformin (in total column of “without metformin”) and significantly increased to 65.11 % in the presence of metformin (in total column of “with metformin”), while in PCOS-induced rats, a fairly significant increase from 57.12 % in metformin-untreated oocytes to 64.28 % in metformin-treated oocytes, (*p* < 0.05). No significant differences in the MII stage within the metformin-treated and -untreated COC groups were seen.

**Table 3:** Nuclear maturation of rat oocytes in control, sham, and PCOS groups in the absence and presence of metformin

Groups	Without metformin				With metformin				
	Control	Sham	PCOS	Total	Control	Sham	PCOS	Total	Total
	*N* (%)	*N* (%)	*N* (%)	*N* (%)	*N* (%)	*N* (%)	*N* (%)	*N* (%)	*N* (%)
GV	4 (13.79)	3 (10.34)	4 (14.28)	11 (12.79)	3 (10.34)	4 (13.79)	3 (10.71)	10 (11.62)	21 (12.20)
GVBD	6 (20.68)	5 (17.24)	5 (17.85)	16 (18.60)	6 (20.68)	6 (20.68)	6 (21.42)^a^	18 (20.93)	34 (19.76)
MI	6 (20.68)	6 (20.68)	5 (17.85)	17 (19.76)	7 (24.13)	7 (24.13)	6 (21.42) ^a^	20 (23.25)	37 (21.51)
A-T	1 (3.44)	3 (10.34)	2 (7.14)	6 (6.97)	1 (3.44)	1 (3.44)	2 (7.14)	4 (4.65)	10 (5.81)
MII	5 (17.24)	5 (17.24)	4 (14.28)	14 (16.27)	5 (17.24)	5 (17.24)	4 (14.28)	14 (16.27)	28 (16.27)
Deg	2 (6.89)	2 (6.89)	3 (10.71)	7 (8.13)	2 (6.89)	1 (3.44)	3 (10.71)	6 (6.97)	13 (7.55)
ND	5 (17.24)	5 (17.24)	5 (17.85	15 (17.44)	5 (17.24)	5 (17.24)	4 (14.28)	14 (16.27)	29 (16.86)
Total	29	29	28	86	29	29	28	86	172

*PCOS* polycystic ovary syndrome; *GV* germinal vesicle; *GVBD* germinal vesicle breakdown; *MI* metaphase I; *A-T* anaphase-telophase; *MII* metaphase II; *Deg.* degenerated; *ND* not-detectable

^a)^Significant differences from without metformin, (*p* < 0.05)

### Light and electron microscope observations

**Fig. 3:**
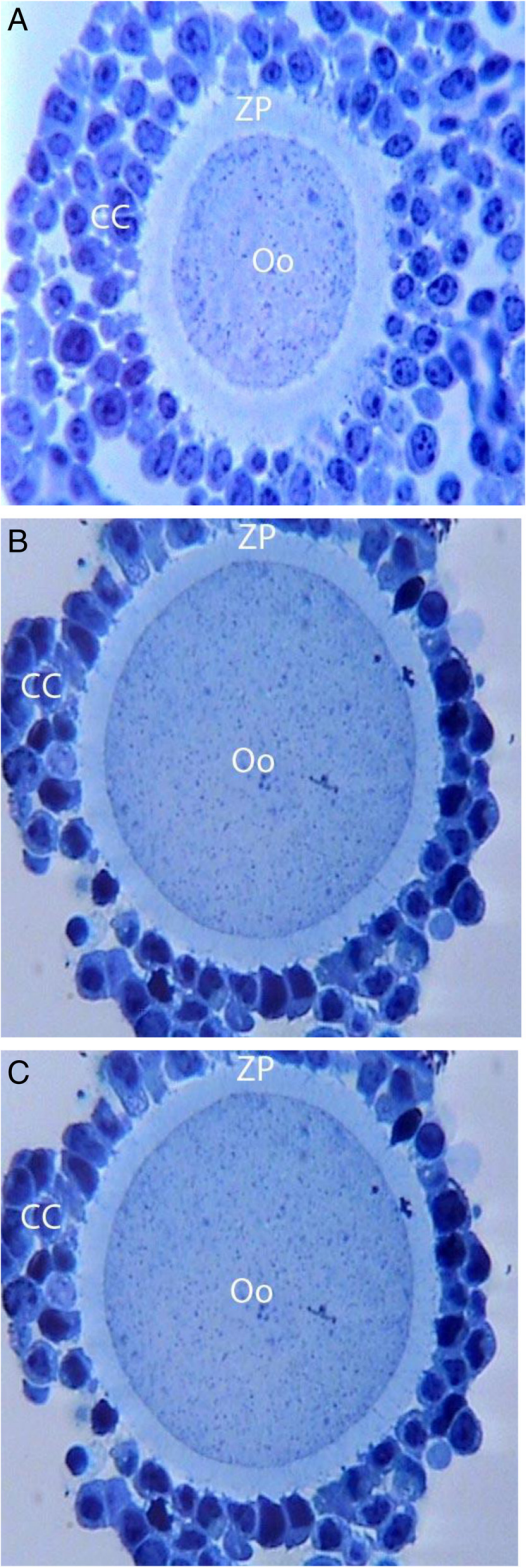
Light micrographs of oocytes cultured for, (**a**) 12 h in the absence of metformin in the normal; (**b**) 24 h in the absence of metformin in the PCOS-induced; (**c**) 36 h in the presence of metformin in the PCOS-induced rats. Few atretic and dark small cumulus cells noticed in (**c**). Resinate 1 μm-sections, toluidine blue staining (400×). ZP, zona pellucida; Oo, oocyte; CC, cumulus cells

**Fig. 4:**
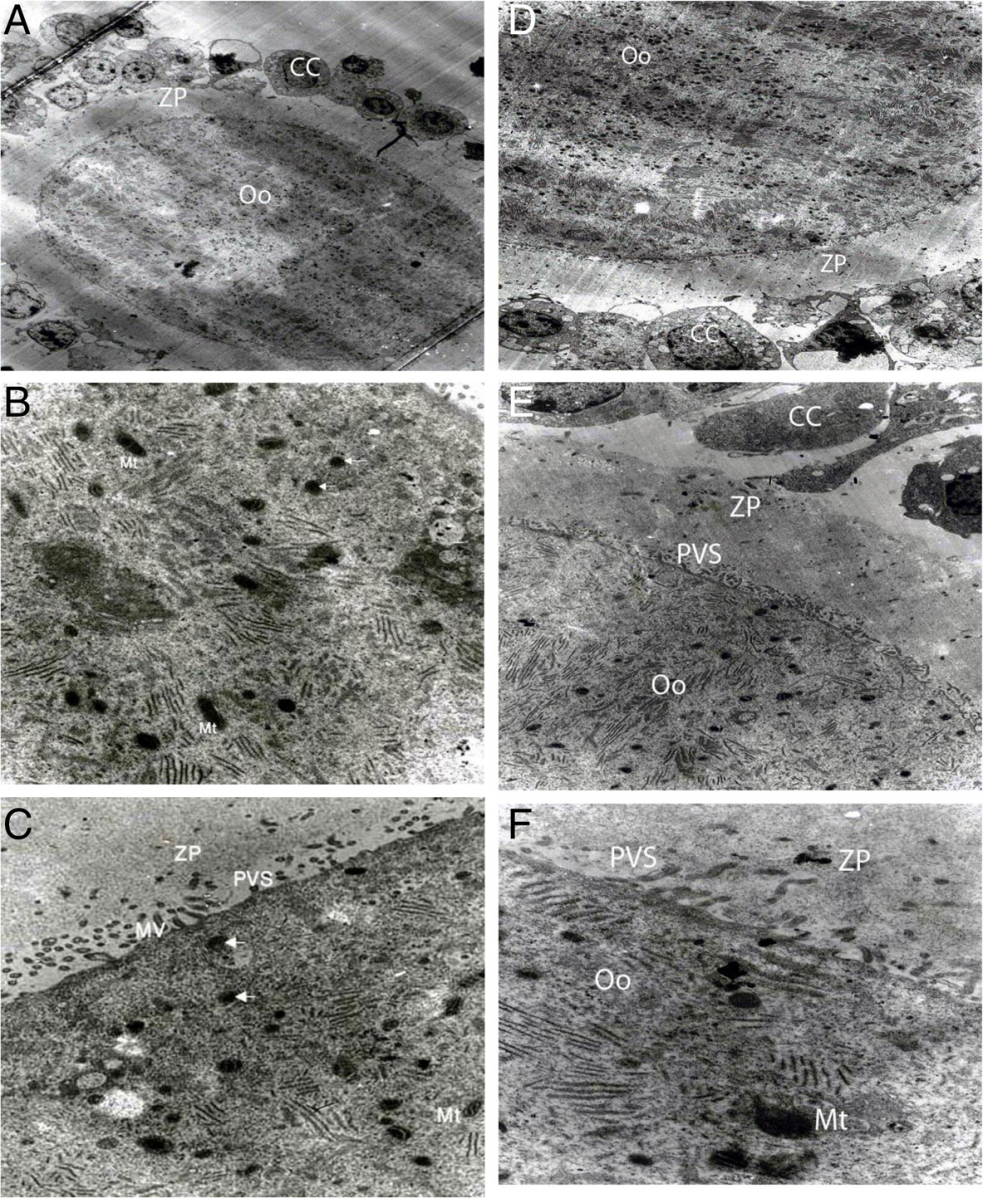
Electron micrographs of oocytes cultured for, (**a**) 12 h in the absence of metformin from the PCOS-induced (1500×); (**b**) 12 h in the presence of metformin from the PCOS-induced, the arrows denote the cortical granules (8900×); (**c**) 24 h in the presence of metformin from the normal, the arrows denote the cortical granules (3200×); (**d**) 24 h in the presence of metformin from the PCOS-induced, degenerated cumulus cells were noticed. (1650×); (**e**) 36 h in the absence of metformin from the normal, degenerated cumulus cells were noticed. (3900×); (**f**) 36 h in the presence of metformin from the normal rats, PVS filled by microvilli. (1150×). ZP, zona pellucida; CC, cumulus cells; Oo, oocyte; Mt, mitochondria; PVS, perivitelline space; MV, microvilli

The observation of semi-thin and ultrathin sections of COCs by a light (Fig. 3a, b, c) and a TEM (Fig. 4a, b, c, d, e, f) at different times in normal and PCOS-induced rats incubated with and without metformin, revealed the following features: a decrease in the number of connections between cumulus cells and each cell dispersed from the other; presence of many small and dark granulosa cells; decreased cytoplasmic projections of cumulus cells and oocytes in the zona pellucida; enlargement of the perivitelline space (PVS), particularly during 12–36 h of incubation; and redistribution of some cytoplasmic organelles, particularly cortical granules which close to the oolemma. However, polar bodies and Golgi complexes were not observed. Degenerated cells were observed in cumulus cell in metformin-treated group and non-uniform ZP, non-obvious oolemma and narrow PVS were noticed in the absence of metformin.

## Discussion

Because of ethical and technical restrictions on human experimentation, researchers have used animal PCOS models. The animal models of PCOS have proven useful in determining the causes of PCOS, to perform preclinical trials, and to examine ovarian morphology, hormonal disorders, and the pathogenesis of anovulation in PCOS. Thus far, a persuasive animal model that can replicate all features of human PCOS has not been established. Among mammals, despite the fact that rhesus monkeys and sheep show major ovarian morphological changes [16], rodents are versatile and a more suitable and unique animal for PCOS models [17, 18]. Compare to rhesus monkeys and sheep, rats and mice are inexpensive, readily available, and easy to handle and maintain [16], they have a short reproductive cycle, short estrous cycles, and a short gestational period [17, 19]. Rodent models of PCOS have shown hyperandrogenism and hormonal alteration; ovarian morphological changes, including the presence of multi-cystic follicles; and metabolic disorders [17]. Rodent models of PCOS can be attained by a variety of methods, including constant exposure to light [20], genetic manipulation [17], and administration of hormones, such as testosterone (T) [17], dihydrotestosterone (DHT) [21], EV [22, 23], and letrozole (a non-steroidal aromatase inhibitor) [21, 24]. Among these hormones, T, DHT, and letrozole show many characteristics of human PCOS, including acyclicity, anovulation, polycystic ovaries, hyperandrogenism, and insulin resistance [17]. These features are dependent on the dose of hormones, menstrual cycle phase and duration of treatment, and the waiting time for induction of PCOS [25]. Several injections and/or gavages of T, DHT, and letrozole are likely to increase the possibility of stress and high mortality rates during the experiment and it is also a time-consuming method. However, after a single injection of the EV, it will take about 8–12 weeks to induce PCOS, therefor the stress of injection is reduced, it is well known that stress causes irregularities in the menstrual cycle. The EV-induced PCOS model displays characteristic morphological alterations in the ovary [26], particularly the multi-cystic follicle; as previously shown [27, 28], and mention shortly in results section, which is typically observed in human PCOS. In addition, the EV-induced PCOS model is used in many laboratories, develops hypertension and increases sympathetic activity [29]; these effects lead to increased blood glucose as in this study and it is utilized for autoimmune disorder responses in PCOS [30]. However, the major restrictions of the EV-induced PCOS model are a lack of exactly endocrine and metabolic features associated with human PCOS [17]. It seems that EV can creates a reversible PCO adult rat model, but not PCOS [16, 31, 32], Gonzalo Cruz et al. established an irreversible PCO model when neonatal rat exposure to EV [33]. Recently, Caldwell et al. have demonstrated that long-term DHT administration in mice imitates an extensive of PCOS features [34].

The results of this present study show that a single intramuscular injection of EV induces experimental PCOS in rats, which can be identified by observing the cystic and atretic follicles as previously indicated in our laboratory [27, 28] and alterations in the serum levels of gonadotropins and increases blood glucose level. In our study, as in the study of Stener-Victorin et al. [29], EV may affect the hypothalamic–pituitary–adrenal axis and the ovary, following a hormonal disorder. Various PCOS animal models have shown dissimilar hormonal changes, but our findings confirm the results of studies by Singh [19], and Stener-Victorin et al. [29], who have reported alterations in the serum levels of gonadotropins.

Obesity is a subordinate symptom of PCOS in women [1]. In this study, weight gain in the PCOS-induced rats was lower than that in the control rats, which is comparable with the results of Stener-Victorin et al. [29]. The administration of EV increases adrenal glucocorticoid production, which enhances lipid metabolism, and leads to a decrease in the body weight [29]. However, the obesity is not always observed in women with PCOS [35, 36]. In addition, the amplification of sympathetic activities in women with PCOS was found to increase lipid metabolism and body activity, and consequently, decreases the body weight [31].

Metformin is an insulin-sensitizing drug [8, 37], usually prescribed in patients with PCOS, to induce ovulation, reduce the symptoms of hyperinsulinemia [8], and improve insulin sensitivity to decrease the serum levels of androgen [37]. It has been reported that metformin contributes to *in vitro* maturation of oocytes, which are collected from patients with PCOS [38], and associated with insulin, but not alone, have beneficial effects on oocyte maturation, oocyte quality and production of embryo [8]. Metformin improves the action of insulin on oocyte glutathione (GSH) content, which knocks out the free radicals in the oocytes, resulting in enhanced oocyte competence [37]. These results suggest that metformin accompany with insulin may increase the cytoplasmic maturity of oocytes during IVM. Mansfield et al. demonstrated that metformin in the culture medium has a direct effect on cumulus and theca cells and mediates enzyme activities for the synthesis of steroid hormones. In addition, metformin has inhibitory effects on progesterone and estradiol production on the *in vitro* culture of granulosa cells; progesterone is the secreted end point in the steroid synthesis pathway in these cells [39]. Progesterone plays a role in bovine oocyte maturation, particularly in cytoplasmic maturation, but represents a different role and is dependent on cells (oocyte and/or cumulus cells) and the cell progesterone receptors [40]. In addition, progesterone induces meiosis resumption in cultured bovine COCs in a concentration-dependent manner [41]. Our data show that metformin does not affect all features of nuclear and/or cytoplasmic maturation. These controversial effects of metformin on nuclear and cytoplasmic maturation lead us to believe that the EV-induced PCOS animal model does not clearly present all the features of hyperandrogenism and hyperglycemia. It has been reported that metformin is more effective in a batch of transgenic PCOS-induced mice that categorized by hyperleptinemia and hyperinsulinemia [12]. The lacks of our study are that we have not measured leptin and insulin levels, but hyperglycemia was observed in the EV-induced PCOS animal model, which it may be due to the increase in sympathetic activity [29]. In the other hand it is believed that patients with the most obvious hyperandrogenism have most benefited from metformin treatment [42], therefore, in our study the metformin inability to show all aspects of *in vitro* oocyte maturation and may be resulted from deficiency of EV to induce PCOS.

It is clearly known that the expansion of cumulus cells of COCs is a criterion for oocyte maturation. Nagyova reviewed the mechanisms involved in ovarian follicular processes, including the expansion of cumulus cells, the hyaluronan synthesis and progesterone production in COCs. The expansion of cumulus cells in mouse, porcine, bovine, and rat depends on a specific factor, “cumulus expansion enabling factor”, which secreted by the oocytes and/or in some mammals, by cumulus cells [43]. It was concluded that optimal cumuli expansion promotes embryonic development in bovine oocytes. It is suggested that glutathione is needed for the expansion of cumulus cells and that hyaluronan accumulates in the expanded cumulus cells. Hyaluronan, which builds up within cumulus cells in porcine COCs during cumuli expansion, disrupts cell junctions of COCs and promotes meiotic resumption in oocytes [44]. Our findings concur with those of previous studies and show that in the presence of metformin in PCOS-induced rats, a higher number of cumulus cells of COCs (53.03 %) are completely expanded as compared to those in the absence of metformin (32.83 %). However, this contradicts the findings of Tosca et al. who concluded that metformin inhibits cumuli expansion and oocyte meiotic resumption in bovine COCs (not denuded oocytes), [45]. These results indicate that the presence of metformin in the culture medium may enhance the expansion of cumulus cells, but not in PCOS-induced rats, because metformin acts first on cumulus cell to dissociate around the oocyte [45]. Despite of metformin has a role on the resumption of meiotic division, a small percentage of oocytes reaches to MII stage. Bilodeau-Goeseels et al. reported that both metformin and aminoimidazole- carboxamide ribofuranoside (AICAR) (activators of AMPK) have inhibitory effects on cumulus cells expansion and nuclear maturation in bovine, but not mouse and are greater in COCs than in denuded oocytes due to the presence of cumulus-oocyte projections [11]. Also Nicolas reported that AMPK has same effects on cumulus cells expansion and nuclear maturation in porcine [46]. Metformin decreased progesterone and estradiol productions *in vitro* in human, rat and bovine granulosa cells [10, 39]. In relevant to the species, different mechanisms involved on estradiol production in granulosa cells and metformin may also reduce steroid levels in granulosa cells from follicular cysts. [10]. Increased estradiol and progesterone concentrations have been reported in the ovarian cyst [47].

Resumption of meiotic division is the other principle of oocyte maturation and takes place largely in IVM. In our study, resumption of meiotic division was slightly increased in the presence of metformin, particularly in the PCOS-induced rats. Although the resumption of oocyte meiotic division is the first step of oocyte maturation, completion of oocyte maturation occurs when the oocyte reaches to the MII stage. In contrast to bovine [45] and porcine [48], which metformin arrested COCs at the GV stage, in our study 65.11 % of COCs were initiated meiotic resumption at the first 6 h of IVM in metformin supplemented medium. While, in PCOS-induced rats a fairly significant increase from 57.12 % in metformin-untreated oocytes to 64.28 % in metformin-treated oocytes. Our data show that, no differences were observed in the percentage of the MII stage between metformin-treated and –untreated oocytes, as reported in bovine [45], these data clearly show that low percentage of oocytes reached to the MII of maturation. Thus, the nuclear maturation of oocytes in the PCOS-induced rats may be not affected by metformin. Tosca et al. reported that metformin decreases the number of cumulus and theca cells to generate steroid hormone genes, thus indirectly promoting the nuclear maturation of oocytes [10]. In contrast to our findings, Bilodeau-Goeseels et al. concluded that metformin activates AMPK, which inhibits GVBD in bovine COCs and denuded oocytes, but enhances oocyte meiotic resumption in mice, which is similar to that observed in the PCOS-induced rats in our study. Several studies also reported that AMPK may have contradictory roles in the management of bovine and murine oocyte maturation; it seems that a different mechanism is used to stimulate AMPK in rodents [11]. The difference between the rate of cumulus cell expansion and nuclear maturation in PCOS-induced rat in presence and absence of metformin, may be as a result in a lack of orchestration between these phenomena.

Cytoplasmic maturation is a criterion for deducing the developmental competence of oocyte maturation *in vitro* and includes numerous morphological and biochemical features. The morphological changes include the reduction of the Golgi complex volume, increase in the number of lipid droplets and cortical granules, and enlargement of PVS [49]. The results of this study at the level of light and electron microscopy show that metformin influences few features of oocyte maturation. Cumulus cells around the oocytes in the metformin-treated group were dark, due to nuclear heterochromatin [50], and had lower cell density than the other groups. However, Nottola et al. [51] demonstrated that the presence of dark cumulus cells is related to the accumulation of lipid droplets in the ooplasm, which shows active steroidogenesis in healthy and mature granulosa cells. Our data show that the number of cell junctions between the other cumulus cells and oocytes were decreased, and these tended to expand. According to the findings of Tosca et al. [10], metformin reduces the production of steroids and enzymes in cumulus cells and consequently, causes a decrease in the number of connections between cumulus cells and oocytes; thus, cumulus cells initiate the expansion. Our findings on the effects of metformin, such as a declining trend of cellular links and redistribution of some cytoplasmic organelles, corroborate the findings of Suzuki et al. [52] with human oocytes and granulosa cells cultured in Ham’s F-10. An increase in the number of apoptotic cells was observed in cumulus cell in the metformin-treated group. Note that a non-uniform zona pellucida, non-obvious oolemma, and narrow PVS were observed in the absence of metformin. It is well known that the enlargement of PVS is a morphological change in matured oocytes [49, 53]. As we have shown, the presence of cortical granules close to the inside of oolemma is a symptom of oocyte maturation, which is required to prevent polyspermy [54].

## Conclusions

This study demonstrated that single dose of EV can creates a reversible PCO adult rat model, but not PCOS, it has no effect on the body weight of rats, but modifies sex hormones and blood glucose level. The results of this study revealed that, metformin enhances the COCs to initiate meiotic resumption at the first 6 h of IVM, but the most of the COCs have never completed meiotic division and the percentage of the MII stage is the same in metformin-treated and –untreated oocytes in EV PCOS-induced rats. In our study the metformin inability to show all aspects of *in vitro* oocyte maturation and may be resulted from deficiency of EV to induce PCOS. The results of this study suggest that further studies including, assessment of glucose; insulin and leptin level; pre-treatment in vivo and deferent concentration of metformin; and spindle assembly detection are needed to evaluate the metabolic features and effects of metformin on oocyte at the molecular and cellular levels throughout the IVM of oocytes retrieved from EV PCOS-induced rat.

## Abbreviations

AICAR: aminoimidazole- carboxamide ribofuranoside
AMPK: adenosine monophosphate-activated protein kinase
A-T: anaphase-telophase
COCs: cumulus oocyte complexes
DHT: dihydrotestosterone
EV: estradiol valerate
GSH: Glutathione
GV: Germinal vesicle
GVBD: Germinal vesicle breakdown
IVM: *in vitro* maturation
PCOS: polycystic ovary syndrome
PVS: perivitelline space
SUMS: Shiraz University of Medical Sciences
T: testosterone
TEM: transmission electron microscopy
